# Supplementary material for: Netcombin: An algorithm for constructing optimal phylogenetic network from rooted triplets
Source: PLoS One. 2020 Sep 18;15(9):e0227842. doi: 10.1371/journal.pone.0227842 (PMC7500971; doi:10.1371/journal.pone.0227842)
Supplement: S4 File — (ZIP) [file pone.0227842.s004.zip › SequenceData-Triplet/sequenceData-10-20-30-0-Triplet/length 300, numseq 40, rate 10^-7/6 seq.pdf]

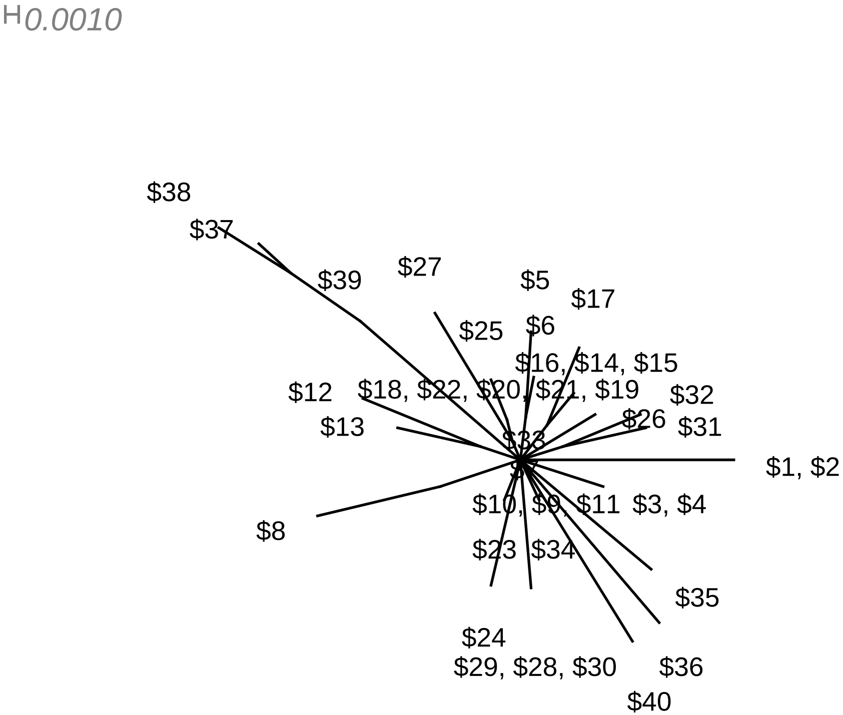
0.0010
$38
$37
$39
$27
$5
$17
$6
$25
$16, $14, $15
$12
$18, $22, $20, $21, $19
$32
$13
$26
$31
$33
$1, $2
$10, $9, $11
$3, $4
$8
$23
$34
$35
$24
$29, $28, $30
$36
$40
